# Transitional Home Care Program Utilizing the Integrated Practice Unit Concept (THC-IPU): Effectiveness in Improving Acute Hospital Utilization

**DOI:** 10.5334/ijic.3050

**Published:** 2017-08-14

**Authors:** Lian Leng Low, Wei Yi Tay, Shu Yun Tan, Elian Hui San Chia, Rachel Marie Towle, Kheng Hock Lee

**Affiliations:** 1 Department of Family Medicine and Continuing Care, Singapore General Hospital, SG; 2 Family Medicine, Duke-NUS Medical School, SG; 3 Health Services Research and Evaluation, Singapore Health Services Regional health System Office, SG

**Keywords:** Home care, transitional care, integrated care, integrated practice unit, readmissions

## Abstract

**Background:**

Organizing care into integrated practice units (IPUs) around conditions and patient segments has been proposed to increase value. We organized transitional care into an IPU (THC-IPU) for a patient segment of functionally dependent patients with limited community ambulation.

**Methods:**

1,166 eligible patients were approached for enrolment into THC-IPU. THC-IPU patients received a comprehensive assessment within two weeks of discharge; medication reconciliation; education using standardized action plans and a dedicated nurse case manager for up to 90 days after discharge. Patients who rejected enrolment into THC-IPU received usual post-discharge care planned by their attending hospital physician, and formed the control group. The primary outcome was the proportion of patients with at least one unscheduled readmission within 30 days after discharge.

**Results:**

We found a statistically significant reduction in 30-day readmissions and emergency department visits in patients on THC-IPU care compared to usual care, even after adjusting for confounders.

**Conclusion:**

Delivering transitional care to patients with functional dependence in the form of home visits and organized into an IPU reduced acute hospital utilization in this patient segment. Extending the program into the pre-hospital discharge phase to include discharge planning can have incremental effectiveness in reducing avoidable hospital readmissions.

## Introduction

Singapore’s population is one of the most rapidly ageing in Asia and an estimated one million or 20% of the country’s population will be 65 years or older by 2030 [1]. Almost a quarter of semi-ambulant and non-ambulant Singapore elderly stay alone or with their elderly spouse [2]. These patients face many challenges after discharge from hospital that increases their risk for unscheduled readmissions. Moreover, primary care and home care in Singapore are relatively lesser developed compared to tertiary care. The misalignment in financial incentives between publicly-funded tertiary care and principally fee for service primary care impedes vertical integration and right-siting of patients across the care continuum. Looking forward, healthcare expenditure is expected to triple from S$4 billion in 2011 to S$12 billion in 2020 [3], driven mainly by inpatient cost. Notwithstanding policy refinement that continue to incentivise care integration, the need to reduce the dependence on high cost hospital-centric care is driving a concerted search for scalable new models of integrated care delivery.

Integrating medical and social care is therefore the foundation to improving health outcomes in the transition period from hospital to home. Care integration is expected to achieve better health outcomes [4,5] and reduce cost by improving coordination of care as exemplified by trials improving the discharge planning process [6] or using advanced practice nurses to provide transitional care [7]. However, many implemented integrated care programs failed to replicate results in clinical trials or rigorously conducted studies, casting reservation on further systemic implementation by policy makers [8,9,10,11]. Therefore, it is of paramount importance that the evaluation and implementation of transitional care programs are reported in literature and centres learn from best practices and evidence.

A review of integrated care literature found that successful programs utilized multi-component interventions [6,11,12] and started early in the care cycle [8,11,13], and concluded that single component interventions are inadequate to meet the challenges during transitions. In Singapore, evidence is emerging for transitional care programs. Successful programs include the national Aged Care Transition (ACTION) program [14] and a home care program that provided transitional care to functionally dependent patients [15]. The ACTION program utilized dedicated care coordinators to provide health coaching and self-management skills to vulnerable older adults. As guidelines instead of specific inclusion criteria was used for recruitment, recruited patients were more heterogeneous as indicated by the range of Charlson Comorbidity Index scores. The home care program showed promising reductions in acute hospital utilization but lacked a comparator group for more rigorous evaluation of its effectiveness [15]. Moreover, shorter term outcomes (i.e. 30-day outcomes) commonly associated with transitional care programs were not reported.

More recently, it has been proposed that care should be organized into integrated practice units (IPUs) around conditions and patient segments for primary and preventive care to provide value-based healthcare [16,17]. Low et al. extended the IPU concept to a virtual ward intervention in a randomized controlled trial for patients at highest risk for readmission that was hugely successful in reducing readmissions, emergency department attendances and hospital length of stay [18]. An IPU contains distinct attributes including organizational structure, responsibility of care, and outcome measures.

To date, no programs utilizing the IPU concept for a patient segment of functionally dependent patients have been described. In this submission, we aim to evaluate the effectiveness of a Transitional Home Care program that applied the IPU concept (THC-IPU) in reducing 30-day readmission for patients with functional dependence admitted to the Singapore General Hospital (SGH). We hypothesize that the THC-IPU program will be effective in reducing the proportion of patients who had a hospital readmission within 30 days of enrolment into the program, as compared to a comparator group of patients who received standard hospital and community care. Additionally, we aim to evaluate the THC-IPU’s effectiveness in improving acute hospital utilization (defined as readmission, emergency department attendance, and specialist outpatient clinic attendances) within 30 days and 90 days of enrolment into the program. Finally, we aim to identify patient subgroups that will benefit maximally from the THC-IPU program.

## Methods

### Study Design, Setting and Population

This was a retrospective cohort study comparing hospital utilization following discharge from an index hospitalization, between an eligible group of patients who accepted an offer of the THC-IPU program at the Singapore General Hospital (SGH) from 1^st^ April 2015 to 31^st^ May 2016, versus the eligible group of patients who did not accept the offer. Patients are considered to be enrolled into the THC-IPU program once the first home visit has been performed. The THC-IPU program is funded by the Ministry of Health (MOH) Singapore since 1^st^ April 2015. However, patients are required to co-pay for the program depending on their means test level. 31^st^ May 2016 was selected as the closing date for this program evaluation as MOH had decided by then to transit to a new model of transitional care and healthcare funding termed as “Hospital to Home” with effect from 1^st^ April 2017. We evaluated patient outcomes until 29^th^ August 2016 (i.e. 90 days after 31^st^ May 2016) for both intervention and control group patients.

SGH is the tertiary hospital in the SingHealth RHS and the site for the THC-IPU program. MOH had created six regional health systems (RHSs) in 2011, each being responsible to integrate care for a specific geographic region in Singapore. The Singapore Health Services (SingHealth) RHS, which is based in the largest healthcare cluster in Singapore, provides care for the Seng Kang area and the South-central part of Singapore.

The inclusion criteria for enrollment into the THC-IPU program are:

- Functional dependence resulting in limited community ambulation.
- Medical care issues requiring follow up in the home setting with or without nursing and social care issues.
- Fit for home discharge after acute hospital stay.

Patients are excluded if they:

- Require end of life palliative care which should be referred to home hospice programs.
- Can access ambulatory care independently or with minimal assistance.

### Intervention

#### THC-IPU team members and their roles

The intervention consisted of a multidisciplinary team of junior physicians, home care nurses, physiotherapist, occupational therapist, speech therapist, pharmacist, medical social worker and administrators organized into an IPU led by senior family physicians (FPs). The IPU is co-located in the same physical locality; share a common electronic patient record and a common mission to reduce avoidable readmissions. FPs in SGH function as community oriented generalists providing general medical care to medical inpatients, including follow-up care of patients in the home care and outpatient specialist clinic upon hospital discharge. The senior FP in the IPU provides supervision to the junior physicians who are typically resident physicians, MOPEX (Medical Officer Posting Exercise) medical officers and clinical associates. The senior FP also attends personally to complicated cases escalated up by the junior physicians and is on standby for subacute home visits. Nurses function as case managers in the THC service. Patient education and care coordination are their part of their core activities. In addition, they can perform nursing procedures i.e. nasogastric tube, urinary catheter insertion when required. Pharmacists assist in medication reconciliation and provide advice on potentially harmful drug adverse events and interactions. The physiotherapist and occupational therapist assess and train patients and their caregivers in mobility and activities of daily living respectively. The speech therapist assess and manage swallowing and communication disorders including training to patients and their caregivers to minimize aspiration risk. Finally, the medical social worker assist in social care arrangements, provide counselling, financial assessments and financial assistance.

#### THC-IPU Intervention Protocol

##### Pre-Hospital Discharge Phase

During the index hospitalization (defined as the hospitalization preceding enrollment into THC-IPU program), patients are managed by their specialists in charge depending on their admitting diagnoses. Potentially eligible patients were identified by the attending team physicians and patient navigators. Patient navigators in SGH function as care coordinators to manage frequent hospital admitters and assist the ward teams in discharge planning. Potentially eligible patients are then referred by the physicians or patient navigators to the THC-IPU program during the hospitalization phase. Patients can also be referred in the immediate post-hospital discharge period if the need became more evident after the hospitalization or patients and caregivers verbalized an inability to cope after hospital discharge. Consequently, the THC-IPU program does not extend into hospitalization phase to provide upstream discharge planning.

##### 1. Comprehensive medical and nursing assessment within two weeks of discharge

The first home visit will be performed by the THC doctor in conjunction with the THC nurse. The THC doctor is a junior physician supervised by a senior family physician. This doctor and nurse team will perform a comprehensive assessment of the patient’s medical and nursing care needs to derive an individualized care plan. In addition, the competency of the care-giver, availability of nursing and home care equipment, adequacy of social support, safety of the home environment and adherence to medication will be assessed to identify social, home environment and functional care issues that can be optimized to support the patient’s care at home. Based on the patient’s medical, functional and social needs, the THC doctor will discuss on subsequent home visits by various team members during the multi-disciplinary team meeting (MDM).

##### 2. Medication Reconciliation

At the home visit, the THC doctor will reconcile all of the participant’s medication regimens to ensure that there are no discrepancies or duplication and to review each medication with the participant and/or their caregiver to ensure that they understand its purpose, instructions and potential adverse effects. Unnecessary medications are discontinued. During the MDM, pharmacists provide further advice on potentially harmful drug adverse events and interactions.

##### 3. Patient and Caregiver Education using standardized action plans and videos

A core component of the THC nurse’s role is in education and coaching of patients and their caregivers on self-management. The nurse will impart knowledge about the causes, consequences, and management of co-morbid conditions to the patient and/or their caregiver by using standardized SingHealth action plans and education videos. Self-management of nasogastric tubes, indwelling urinary catheters will also be taught. In addition, the nurse will review potential red flags with the patient and/or caregiver that may indicate a worsening of their present state (e.g. fluid overload) or complications (e.g. infections).

##### 4. Setting up a Personal Health Record

The personal health record is a patient-centered document that consists of the core data elements needed to facilitate continuity of the care plan across settings. The core data elements include an active problem list, active medications and hospital appointments, and a list of red flags that correspond to a worsening of the patient’s current state or co-morbidities. Finally, the personal health record will include contact information of the THC nurse for the patient to contact in the event of emergencies. The patient and caregiver will be encouraged to maintain and to continually update the personal health record and to share this document with practitioners across health care settings.

##### 5. Multi-disciplinary Team Meetings (MDMs)

Following the first home visit, the THC team members will discuss all new cases during the weekly MDMs to set goals to be achieved for the patient in next three months. The THC doctors and nurses will assign therapists and medical social worker to cases with functional, home environment and social care issues that can be optimized. The pharmacist will add further input to the medication reconciliation.

##### 6. Dedicated nurse to do telephone follow up to maintain continuity of care

Scheduled calls to check in on the patients are made once a week. During each call, the nurse will review the patient’s progress towards goals established during the initial home visit, discuss any encounters that may have taken place with other health care professionals and review any potential red flags. Throughout the intervention period, the nurses are contactable during office hours. Each nurse was responsible for an average of 30 patients at any time during the program.

##### 7. Allied Health Services

Physiotherapy, occupational therapy, speech therapy and medical social workers are an integral part of the THC-IPU program. They participate in weekly MDM discussions on patients requiring allied health services in the transition period. Functional outcomes are tracked using the modified Barthel index by the therapists and not the focus of this study.

##### 8. Subsequent Routine or Subacute home visits

During a planned routine visit, the doctor will review the goals set during the initial home visit, the competency of the care-giver, adherence to medication, the necessary nursing and home care equipment has been obtained, and the home environment has been optimized.

Patients and/or their caregivers may contact the THC team to perform subacute visits when there is destabilization in the medical conditions. The THC nurse will triage the case to determine if a sub-acute visit is indicated. The team will perform a subacute visit within 48 hours and start treatment promptly to prevent an avoidable readmission.

##### 9. Readmissions Review

All readmissions back to SGH will be reviewed by the THC doctor and nurse to facilitate discharge planning and early discharge whenever possible. All readmitted cases are reviewed for preventable causes. The team will perform a home visit within two weeks of discharge from the readmission.

Patients are discharged from the THC-IPU program at the end of three months or earlier if their health status is stable and can be transited to a suitable community care provider.

##### Control group

Patients who were assessed to be eligible for the THC-IPU program but declined to be enrolled when approached constitute the control group for this study. Common reasons for rejecting the THC-IPU program include cost, perceived lack of need and availability of pre-existing care providers. The control group received hospital standard of care when they are hospitalized. Patients are managed by their specialists in charge depending on their admitting diagnoses. Patients may be referred to various community services on discharge if deemed necessary by their primary team physicians. Continuing care post-discharge may be provided at the specialist outpatient clinics or a primary care provider identified by the primary team. The THC-IPU program would not be available for control group patients.

##### Outcomes

The primary outcome measure in this study is the proportion of patients who had a readmission within 30-days of enrollment into THC-IPU program. Secondary outcomes include the proportion of patients who had a readmission within 90 days of enrollment, and proportion of patients who had an emergency department (ED) attendance within 30 and 90 days of enrollment. As control group patients do not have an enrollment date, we used the index discharge date for comparison.

##### Statistical Analysis

We compared the baseline socio-demographics and acute hospital utilization rates between the control and intervention group using Chi-square for categorical variables and 2-sample t-tests for continuous variables. We also tested the differences in the continuous variables using the nonparametric Wilcoxon Rank Sum test because most of the variables were not approximately normal distributed. We reported the results from the parametric t-test when it is consistent with the nonparametric test and the results from the nonparametric Wilcoxon Rank Sum test when it is not.

To examine the effectiveness of the program in reducing the proportion of patients who had a hospital readmission, ED attendance, and specialist outpatient clinic attendances within 30 days of enrolment into the program, we applied a backward stepwise logistic regression model where the initial full model included all available baseline socio-demographic data and acute hospital utilization rates as independent variables in addition to the main predictor of treatment group. The independent variable with the largest p-value > 0.05 (except for treatment group) is removed from the model one by one until all the independent variables left in the model are significant. The stepwise procedure was repeated twice by varying the independent variables included in the initial model. We assessed each final model using the Hosmer-Lemeshow goodness-of-fit test and the Aikaike Information Criteria (AIC). The model which fits the observed data well and has the lowest AIC is selected as the best model. We presented the adjusted odds ratio for the intervention group as compared to the control group from the selected model.

We also conducted subgroup analyses to study the effectiveness of the program for different patient subgroups. We tested the significance of the interaction between treatment group and all other baseline variables to determine whether the odds ratio of having a 30 day admission/ED attendance/Specialist Outpatient Clinic (SOC) attendance vary significantly among subgroups of patients. With 21 interaction terms and three outcome measures, we could expect up to three statistically significant results (p < 0.05) due to chance. We reported the odds ratio and 95% confidence intervals for the various subgroups of significant baseline characteristics. All analyses were performed on STATA/IC 13.1.

### Results

#### Patient Baseline Characteristics and Acute Hospital Utilization Rates

A total of 1,166 patients were included in the analysis, of which 625 were in the control group and 541 in the intervention group. Overall, the patients were elderly with a mean age of 79.1 years (standard deviation 11.1). There were more females than males (63.0% vs 37.0%) and a large majority of Chinese ethnicity (81.8%). More than a quarter of the patients lived in rental flats (27.4%). The severity of the patients’ condition at baseline was measured using the Charlson Comorbidity Index and the median score was 3 and ranges from 0 to 20. The median length of stay (LOS) of the patients’ index hospitalization is 6 days and ranges from 1 to 91.

The control and intervention group are comparable in terms of age and gender distribution (Table 1). There are some differences in the distribution of the ethnicity group (p = 0.001) and the proportion of patients living in rental flats (p = 0.041). Patients in the intervention group also have a significantly higher (p < 0.001) mean Charlson score of 3.7 (SD 2.8) as compared to patients in the control group with mean 2.8 (SD 2.3). The number of admissions and ED attendances in periods up to a year before index hospitalization are mostly comparable with the exception of 30 days pre-index where patients in intervention group have fewer admissions and ED attendances compared to the control group (p < 0.001 for both). Conversely, patients in the control group have fewer SOC attendances than patients in the intervention group for periods of 1 year, 180 days, 90 days and 30 days before index utilization (p = 0.026, 0.017, 0.017, 0.035 respectively).

**Table 1:** Baseline Patient Demographics and Acute Hospital Utilization Rates.

Characteristics	Control (*n* = 625)	Intervention (*n* = 541)	p-value

Age, mean (SD)	79.0 (10.8)	79.3 (11.2)	0.627
Gender, n (%)			
Female	392 (62.7%)	342 (63.2%)	0.861
Male	233 (37.3%)	199 (36.8%)	
Ethnicity, n (%)			
Chinese	525 (84.0%)	429 (79.3%)	0.001
Malay	43 (6.9%)	71 (13.1%)	
Indian	41 (6.6%)	36 (6.7%)	
Others	16 (2.6%)	5 (0.9%)	
Rental Housing Status, n (%)			
No	469 (75.0%)	377 (69.7%)	0.041
Yes	156 (25.0%)	164 (30.3%)	
Charlson Comorbidity Index Score, mean (SD)	2.8 (2.3)	3.7 (2.8)	<0.001
Admit Source Type, n (%)			
A&E	571 (91.4%)	503 (93.0%)	0.307
Non A&E	54 (8.6%)	38 (7.0%)	
Admit Type, n (%)			
Emergency	571 (91.4%)	502 (92.8%)	0.368
Non-Emergency	54 (8.6%)	39 (7.2%)	
Admit Class, n (%)			
Class A	22 (3.5%)	9 (1.7%)	0.051
Class B1	30 (4.8%)	15 (2.8%)	
Class B2	299 (47.8%)	259 (47.9%)	
Class C	274 (43.8%)	258 (47.7%)	
Length of Stay (LOS), mean (SD)	11.2 (13.2)	10.3 (12.9)	0.209
No of admissions			
1 year before index admission/THC start date, mean (SD)	1.5 (1.9)	1.7 (2.0)	0.183
180 days before index admission/THC start date, median (range)	1 (0–7)	1 (0–8)	0.116
90 days before index admission/THC start date, mean (SD)	0.5 (0.9)	0.6 (0.8)	0.502
30 days before index admission/THC start date, mean (SD)	0.2 (0.6)	0.1 (0.4)	<0.001
No of ED visits			
1 year before index admission/THC start date, mean (SD)	2.6 (2.3)	2.7 (2.2)	0.252
180 days before index admission/THC start date, mean (SD)	1.9 (1.6)	2.0 (1.5)	0.337
90 days before index admission/THC start date, mean (SD)	1.5 (1.0)	1.5 (1.0)	0.369
30 days before index admission/THC start date, mean (SD)	1.2 (0.7)	0.8 (0.7)	<0.001
No of SOC visits			
1 year before index admission/THC start date, mean (SD)	4.7 (6.7)	5.7 (7.7)	0.026
180 days before index admission/THC start date, mean (SD)	2.4 (3.6)	2.9 (4.0)	0.017
90 days before index admission/THC start date, mean (SD)	1.3 (2.1)	1.6 (2.2)	0.017
30 days before index admission/THC start date, mean (SD)	0.5 (0.9)	0.6 (1.0)	0.035

SD: standard deviation; ED: emergency department; SOC: Specialist Outpatient Clinic; p < 0.05 (Statistically significant).

#### Effectiveness of the THC-IPU Program

The percentage of patients within the intervention group having to utilize hospital resources within 30 days of index hospitalization is lower than the percentage of patients within the control group (Table 2). The differences in percentage between control and intervention for admission (19.2% vs 13.1%) and ED attendances (19.8% vs 15.2%) are statistically significant (p = 0.005 and 0.037 respectively) whereas the differences for SOC attendances (45.8% vs 43.3%) is not statistically significant (p = 0.390).

**Table 2:** Overall difference in the proportion of patients with hospital utilization within 30 days after index hospitalization.

Outcome Measures	Control *n* = 625	Intervention *n* = 541	p-value

Admissions, n (%)	120 (19.2%)	71 (13.1%)	0.005
ED attendances, n (%)	124 (19.8%)	82 (15.2%)	0.037
SOC attendances, n (%)	286 (45.8%)	234 (43.3%)	0.390

ED: emergency department; SOC: Specialist Outpatient Clinic; p < 0.05 (Statistically significant).

The results are consistent after using the model building approach to adjust for confounders (Table 3). Patients in the intervention group are 38% less likely to have an admission 30 days after enrolment compared to the control group (adjusted odds ratio 0.62; 95% confidence interval 0.44 – 0.86; p = 0.004). For ED attendances, they are also 33% less likely than patients in control group with an adjusted odds ratio of 0.67 (95% CI 0.49 – 0.92; p = 0.013). Although not statistically significant (p = 0.090), patients in the intervention group are 19% less likely to have an SOC attendance 30 days after enrolment compared to the control group (aOR 0.81; 95% CI 0.63 – 1.03).

**Table 3:** Odds Ratio from Stepwise Multiple Logistic Regression Modeling, adjusted for other significant predictors.

Outcome Measures	Intervention group (Reference: Control group)	

	Adjusted Odds Ratio (95% CI)	p-value

30 days readmission*	0.62 (0.44–0.86)	0.004
30 days ED attendance	0.67 (0.49–0.92)	0.013
30 days SOC attendance	0.81 (0.63–1.03)	0.090
90 days readmission	0.72 (0.55–0.93)	0.012
90 days ED attendance	0.76 (0.59–0.98)	0.037
90 days SOC attendance	0.86 (0.66–1.13)	0.291

*: Primary outcome measure; ED: emergency department; SOC: Specialist Outpatient Clinic; p < 0.05 (Statistically significant).

#### Subgroup Analyses for Admission and ED Attendances

There were six interaction terms found to be statistically significant (p < 0.05), of which two were for the outcome of 30 days SOC attendances. As the overall difference for SOC attendances is not statistically significant, we did not present the results here.

For 30 days admission, there were significant differences in the odds ratio comparing intervention to control (p = 0.009) between different levels of patients’ Charlson Score at baseline (Table 4). Patients with higher Charlson Score have lower odds ratios than patients with Charlson Score of 0 to 2 (OR 0.93; 95% CI 0.55 – 1.54). Patients in both subgroups of Charlson Score 3 to 5 and Charlson Score more than 5 have significant odds ratio of 0.46 (95% CI 0.26 – 0.81) and 0.43 (95% CI 0.20 – 0.93) respectively.

**Table 4:** Subgroup analyses for Admission and ED attendance within 30 days.

Outcome: Admission within 30 days	Control *n* = 625	Intervention *n* = 541	Odds Ratio (95% CI)	p-value for interaction

Subgroup: Charlson Score				
Charlson Score: 0–2	51 (15.6%)	31 (14.6%)	0.93 (0.55–1.54)	0.009
Charlson Score: 3–5	46 (21.7%)	24 (11.3%)	0.46 (0.26–0.81)	
Charlson Score >5	23 (27.1%)	16 (13.8%)	0.43 (0.20–0.93)	
**Outcome: A&E attendances within 30 days**	**Control** ***n* = 625**	**Intervention** ***n* = 541**	**Odds Ratio** **(95% CI)**	**p-value for interaction**

Subgroup: Gender				
Female	87 (22.2%)	44 (12.9%)	0.52 (0.34–0.78)	0.007
Male	37 (15.9%)	38 (19.1%)	1.25 (0.74–2.12)	
Subgroup: Charlson Score				
Charlson Score: 0–2	58 (17.7%)	36 (16.9%)	0.95 (0.58–1.53)	0.047
Charlson Score: 3–5	42 (19.8%)	27 (12.7%)	0.59 (0.33–1.03)	
Charlson Score > 5	24 (28.2%)	19 (16.4%)	0.50 (0.24–1.04)	
Subgroup: No. of admissions 30 days before				
0 admissions	95 (18.7%)	60 (12.6%)	0.63 (0.43–0.90)	0.024
1 or more admissions	29 (24.8%)	22 (33.3%)	1.52 (0.74–3.09)	

There were three significant interaction terms for the outcome of ED attendances, namely for different levels of number of admissions 30 days before index hospitalization (p = 0.024), Charlson Score (p = 0.047), and gender (p = 0.007) (Table 4). Patients with no admissions 30 days before index hospitalization have lower odds ratio (OR 0.63; 95% CI 0.43 – 0.90) than patients with 1 or more admissions (OR 1.52; 95% CI 0.74 – 3.09).

Similar to the 30 days admission outcome, we also found that the odds ratios for patients with Charlson Score 3 to 5 (OR 0.59; 95% CI 0.33 – 1.03) and Charlson Score more than 5 (OR 0.50; 95% CI 0.24 – 1.04) are lower than patients with the smaller Charlson Score of 0 to 2 (OR 0.95; 95% CI 0.58 – 1.53). Females also have a lower odds ratio (OR 0.52; 95% CI 0.34 – 0.78) than males (OR 1.25; 95% CI 0.74 – 2.12).

## Discussion

Our study showed that the THC-IPU program was associated with reduced likelihood of hospital readmission and ED attendance rates at 30 days and up till 90 days after hospital discharge, which is suggestive of a positive contribution from transitional care organized as an integrated practice unit. For both outcomes of 30-day hospital readmissions and ED attendances, subgroup analyses suggest that THC-IPU program was associated with reduced likelihood in patients with a Charlson score of 3 or more. This is consistent with literature that transitional care interventions should be targeted at patients of sufficiently high risk to benefit [11].

The effectiveness of a transitional care program is dependent on its intensity and duration [19]. Evidence suggest that high intensity transitional care intervention up to a duration of six months, is effective in reducing short term (30 days), intermediate term (31–180 days) and even long term (181–365 days) hospital readmissions [19,20]. For high intensity transitional care interventions to be effective they should be multicomponent [8,11,13,21,22] and include at least care coordination by a nurse, communication between the discharging physician and the primary care provider as well as a home visit within three days of discharge [19]. The THC-IPU care bundle is considered a high intensity intervention as it includes the following components: case management and care coordination by nurses, home visit within two weeks of discharge, telephone follow up, self-management education and caregiver involvement. THC-IPU program could have prevented more hospital readmissions if home visits could be done earlier, within days of discharge.

Studies have suggested that interventions that start prior to and continue after discharge are more effective in reducing hospital readmissions [23]. Despite lacking the pre-discharge component, the THC-IPU program was effective in reducing short term hospital readmissions. This gap is made up by enhanced communication between the discharging hospital team and the THC-IPU team, as both teams are from the same hospital organization. Access to electronic hospital records allowed for seamless transition of care, and enabling the multi-disciplinary THC-IPU team to function effectively as the “primary care provider” and the single point of contact looking after the patients during the 3 months of transitional care. It is likely that the THC-IPU program could have been more effective if it extended upstream to include interventions such as hospital discharge planning and early patient education and activation [23].

The THC-IPU program was associated with better outcomes in patients who are at higher risk of hospital readmission. This trend is also evident in other studies where patients who are more frail and with multiple comorbidities and functional disabilities benefitted most from home based interventions [24]. This suggests that the THC-IPU program is effective in targeting the intended population and hence should be offered to a selected population of high risk patients. The THC-IPU program was however, not effective in reducing SOC attendances. There is currently a lack of transitional care literature focusing on outpatient clinic attendances as an outcome. It is likely that the THC-IPU program was not adequately active in consolidating the patients’ SOC attendances due to hesitancies in re-arranging appointments. Moreover, many patients will require continuing SOC follow up for their organ diseases after the completion of the 3 month THC-IPU program.

Although we had carefully planned for the evaluation of the THC-IPU program, a randomized controlled trial study design was not feasible due to the service obligations of the hospital. It is possible and perhaps likely that patients who rejected to be enrolled into the THC-IPU program are not entirely comparable with patients who enrolled into the THC-IPU. However, we minimized such selection bias by collecting known confounders for 30-day readmission risk [25,26], and adjusted for these confounders in our stepwise logistic regression model.

## Conclusion

Our study showed that delivering transitional care to a patient segment with functional dependence was associated with reduced likelihood of hospital readmission and ED attendance rates in this group of patients who might otherwise face difficulties in returning to hospital for follow ups. This is suggestive of a positive contribution in the form of home visits and organization of care as an IPU. Extending the program into the pre-hospital discharge phase to include discharge planning is likely to have incremental effectiveness in reducing avoidable hospital readmissions.
